# A conservative treatment for chronic obstructive sialoadenitis by intraductal instillation of mucolytic, steroids and antibiotic solution

**DOI:** 10.1007/s00405-021-06930-2

**Published:** 2021-06-10

**Authors:** Francesco Lorusso, Angelo Immordino, Francesco Dispenza, Federico Sireci, Salvatore Gallina

**Affiliations:** 1 Unit of Otorhinolaringology, Azienda Ospedaliera Universitaria Policlinico “Paolo Giaccone”, University of Palermo, Via del Vespro, 133, 90127 Palermo, Italy; 2 Unit of Otorhinolaringology, Department of Biomedicina, Neuroscienze E Diagnostica Avanzata, Azienda Ospedaliera Universitaria Policlinico “Paolo Giaccone”, University of Palermo, Via del Vespro, 133, 90127 Palermo, Italy

**Keywords:** Sialadenitis, Chronic obstructive sialadenitis, Intraductal instillation, Sialoendoscopy

## Abstract

**Purposes:**

Reporting our experience in treating chronic obstructive sialadenitis with a protocol consisting of sialoendoscopy and intraductal instillation of antibiotics, steroids and *n*-acetyl-cysteine (NAC) solution.

**Methods:**

Prospective study of patients with chronic obstructive sialadenitis with no apparent lithiasic obstructions, with recurrent non-lithiasic sialoadenitis and patients with lithiasic sialoadenitis not solved with sialoendoscopy. In all cases, a sialoendoscopy was performed. All the patients affected by lithiasic sialoadenitis where the chronic inflammation was resolved with sialoendoscopy were excluded from the study. The mid-term follow-up was performed at 12 months via phone interview, to understand whether patients had developed any further symptoms after the treatment.

**Results:**

This study included 26 patients. All the patient without sialolithiasis have not reported any symptoms during the follow-up period. Two of those with sialolithiasis have not shown any signs of recurrence. The remaining three patients with non-resolved sialolithiasis had a recurrence of symptoms which were treated again with 1 intraductal administration of betamethasone, gentamicine and NAC, showing immediately a regression of the symptoms.

**Conclusions:**

Intraductal administration of gentamicin + NAC + betamethasone seemed effective for the therapy of chronic obstructive sialoadenitis. Our protocol seemed effective also in that cases where it was not possible to remove or detect endoscopically an obstruction. In all these cases we have noticed an increase in the symptom-free time even in cases where it was not possible to remove the stones.

## Introduction

Chronic sialadenitis is a localized condition of the salivary gland characterized by repeated episodes of swelling, pain and inflammation. The trigger factor is usually salivary duct obstruction, resulting in salivary stasis, which predisposes the patient to recurrent episodes of infection and inflammation.

Sialolithiasis represents the most common cause of ductal obstruction, followed by mucus plugs deposition, stenosis of the ducts, neoplastic extrinsic compression, congenital dilatation and foreign bodies [1].

Clinically, chronic sialadenitis is characterized by recurrent episodes of sudden swelling of the affected salivary gland, associated with food intake. The skin surface may appear normal or slightly inflamed with varying degrees of tenderness. Patients frequently report an initial episode of acute suppurative sialadenitis, followed by silent periods which change over time. Salivary flow is markedly reduced and usually secretion is viscous and milky.

Diagnostic procedures include traditionally imaging such as plain radiographs, ultrasound, sialography, scintigraphy, CT and MRI [2]. MRI is the gold standard for showing soft tissue [3].In particular the sialo-MRI shows the parenchymal tissues with high accuracy, depending on the salivary flow which acts as “contrast medium” and therefore might impair the examination [4, 5] if reduced. Imaging often underestimates duct obstruction [6]. An effective method to evaluate the ductal system is sialoendoscopy, which allows the assessment of the state of the duct and possible causes of obstruction.

Several treatments are available which include: intraoral or external removal of the stone, if present, extracorporeal or intracorporeal litotripsy, oral antibiotics and oral anti-inflammatory, intraductal instillations of saline soluctions, steroids or antibiotics, methyl violet (1%) injection to produce gland atrophy, parasympathectomy, kallikrein inhibitor, ductal ligation, superficial or total parotidectomy.

We report our experience in treating obstructive chronic sialadenitis with sialoendoscopy and a pharmaceutical protocol consisting of intraductal instillation of antibiotics, steroids and NAC which, as several studies have highlighted, have an inhibiting and degrading action on the extracellular polysaccharides (EPS) that forms the biofilm, allowing the antibiotic to reach the bacteria.

## Methods

Between October 2009 and June 2019, at the Department of Otorhinolaryngology of the University of Palermo, we treated 140 patients with chronic obstructive sialadenitis.

The following categories of patients were enrolled in this study: patients with no apparent lithiasic obstructions, according to imaging studies and sialoendoscopy; patients with recurrent non-lithiasic sialoadenitis and patients with lithiasic sialoadenitis not solved with sialoendoscopy. All the patients affected by lithiasic sialoadenitis where the obstruction was resolved with sialoendoscopy were excluded from the study.

Out of 140 patients, 26 patients were enrolled in the study and the remaining 114 were excluded. The diagnosis was made based on medical history, clinical examination and imaging.

The symptoms included recurrent swelling often related to meals, discomfort and, in some cases, pain or burning. Affected glands were enlarged with stretched-elastic consistency and minimal saliva. In most cases, whitish flocculent and jelly-like saliva, from the duct orifice was reported.

In all cases, a sialoendoscopy was performed under local anesthesia with an injection of mepivacaine in the duct. The papilla was expanded by dilatators of increasing diameters. This procedure has allowed us to explore the ductal system thoroughly, displaying the state of the walls of the ducts and the presence of mucous plugs or stones, probably with higher accuracy than imaging, as evidenced by Nahlieli and Baruchin’s [7]. When stones or mucous precipitate was identified, the removal was facilitated by irrigation with a corticosteroid solution and the stones were removed with Dormia basket. In five cases the localization of the stones in the most distal branches did not allow their removal.

In all the 26 selected cases with persistent obstruction, 3 days after the sialoendoscopy we instilled intra-ductally a 5 ml solution composed by 1 ml betamethasone (4 mg/1 ml) + 2 ml gentamicine (80 mg/2 ml) + 2 ml NAC (300 mg/3 ml).

The orifice of the duct was enlarged by a probe and then the duct cannulated with a polyethylene catheter, i.e. No. 20 and No. 22 for the submandibular gland and for the parotid, respectively. Catheter was kept in place for 15 min to prevent reflux. After the procedure was observed a minimal discomfort and enlargement of the gland for about a day. The procedure was repeated depending on the therapeutic response every 3 days for 4 or 5 times a day. In addition, between the treatments we invited the patient to eat salivary-activating foods (e.g. orange, lemon drops, vinegar, etc.) to facilitate the recovery of the salivary flow, discharge of intraductal precipitates and clearing of the ducts.

Follow-up was performed via phone interview, to understand whether patients had developed any further symptoms after the treatment.

The present study has some limitations related to the lack of a control group. Therefore, we could not compare patients treated according to our protocol (i.e. betamethasone + gentamicine + NAC) with patients treated with steroid intraductal irrigation only.

## Results

All the 26 patient-reported variable recurrent submandibular or parotid swelling over a period of 2 months up to 5 years, with one case reporting symptoms for 20 years.

They ranged in age from 39 to 59 years, with 16 being men and 10 women. Eleven patients had a chronic parotitis and 15 had a chronic submandibular sialadenitis. Out of these 15, five were patients with submandibular sialolithiasis where it was not possible to remove the stone with sialoendoscopy or any other minimally invasive approaches.

In 8 cases a pretreatment therapy was performed with an oral antibiotic, associated either with corticosteroid therapy, which, however, did not bring lasting benefits.

After sialoendoscopy and the first intraductal steroid irrigation was reported the discharge of white and flocculent lumpy material from the duct.

The first week after sialoendoscopy was generally caratherized by moderate swelling and discomfort of the affected gland.

Three days after the sialoendoscopy, intraductal irrigation was performed and repeated every 3 days for 4 or 5 times a day with a 5 ml solution composed of 1 ml betamethasone (4 mg/1 ml) + 2 ml gentamicine (80 mg/2 ml) + 2 ml NAC (300 mg/3 ml). The complete normalization of the appearance of saliva was reported after 2 or 3 intraductal applications which, together with the disappearance of the swelling and discomfort, defines the clinical cure.

The mid-term follow-up was performed at 12 months. All the patients without sialolithiasis (80.8%) have not reported any symptoms during the follow-up period. Two of those with sialolithiasis (7.7%) have not shown any signs recurrence. The remaining three patients with non-resolved sialolithiasis (11.5%) had a recurrence of symptoms which were treated again with one intraductal administration of betamethasone, gentamicine and NAC, showing immediately a regression of the symptoms.

## Discussion

Treatment of chronic sialadenitis should focus on inflammation, ductal obstruction and chronic infection often associated.

Short-term systemic steroid therapy can be used to reduce the gland inflammation and, as demonstrated by Baurmash [8], to restore the normal blood exocrine gland permeability barriers. The restoration of the correct glandular permeability would reduce the exudation of serum proteins (i.e. IgA,IgG,IgM and albumin) and lactoferrin preventing their coagulation and precipitation due to the low PH and stasis which may cause further intraductal accumulation and ductal obstruction.

Irrigation with intraductal steroids seems to be a better therapeutic solution compared to systemic steroid especially in patients with comorbidity such as hypertension and diabetes. Intraductal steroids are in fact capable of controlling the inflammatory component of the pathology by reducing the associated symptomatology with no appearance of side effects [9]. The good result of intraductal irrigation with steroids is also confirmed by Capaccio et al. [10].

The reduction of gland inflammation alone without removal of the obstruction is not sufficient to solve the swelling. Obstructive sialoadenitis in most cases are caused by sialoliths. We know that submandibular gland is the most commonly affected. As a matter of fact 80% to 90% of stones develop in Wharton’s duct, 10% to 20% in the Stensen’s duct and 1% in the sublingual duct [11], with the highest incidence in the fifth and eighth decade. The composition of stones is predominantly inorganic, with calcium phosphate and carbonate and a small amount of other salts in combination with an organic matrix of glycoproteins and mucopolysaccharides [12]. The exact etiology of sialolithiasis is uncertain, but salivary stasis, ductal inflammation and injury seem to be the most important risk factors. These factors result in an alteration of the mucoid element of saliva which leads to an organic gel, that becomes the framework for the deposition of salts. The insufficient salivary flow facilitates ascending salivary duct infections through the oral cavity. Bacterial infection, production of pus and perpetuation of inflammation create the appropriate environment for the multiplication of the stones.

There are many disputes around inflammation versus infection as the primary cause of the condition. Williams et al. support a different relationship between sialolithiasis and sialoadenitis mechanisms if this occur in submandibular or parotid gland [13]. According to this evidence, in the submandibular gland, the development of sialolithiasis might be the primary event that results in stagnation of saliva and inflammation, encouraging bacterial migration and resulting in sialoadenitis. In parotid gland inflammation instead a ductal injury caused by chronic sialadenitis seems to be the initiating process for sialolithiasis.

Ascending infections that occur due to the reduced salivary flow are caused by bacteria commonly found as normal microbial flora of the upper airways. Bacteria find a favorable environment for their growth and cause infections that can give acute manifestations such as purulent sialadenitis or chronic manifestations related to the persistence of bacteria in the salivary ducts often stick to the surface of the stones and protected by biofilm.

In ascending infections, which can be caused by a salivary stasis that determines recurrent chronic sialadenitis in a vicious circle, an important role seems to be played by the development of bacterial biofilms, involved in at least 60% of all chronic infections and/or relapsing. The main characteristic of biofilms is the ability to make microorganisms more resistant to exogenous aggressions. Only antibiotics are in fact effective on planktonic pathogens, responsible for the acute clinical manifestations of the infections. However, a reservoir of protected bacteria remains in the biofilm which acts as the primary source of new waves of planktonic organisms capable of giving rise to acute exacerbations [14].

The most frequently found bacteria are *S. aureus*, *S. pyogenes*, *S. viridans*, *S. pneumoniae*, *H. influenzae*, *M. catarrhalis* and *P. aeruginosa*. Most of these bacteria, in suitable environmental conditions such as those created by salivary stasis, can create, through the formation of an extracellular polysaccharide matrix, a biofilm that consolidates its adhesion and protects them both from immune responses and antibiotics, also contributing to the complete obstruction of the salivary ducts [11, 15–17]. Biofilms are well-structured groups of bacteria and eukaryotic cells, enclosed in a polymeric matrix produced by the cells themselves, capable of adhering to both inert and non-inert surfaces. After bacterial adhesion, the exopolysaccharide polymers of the glycocalyx are produced which, melting in a matrix, form the biofilm [18]. Biofilm-equipped bacteria are much more resistant to antibiotics than their planktonic counterpart even up to 1000 times, according to some authors [15].

This condition requires the use of innovative strategies for the treatment and prevention of infections associated with biofilm. Particular interest aroused the study in which the effect on the biofilm and bacterial viability of the association of *N*-acetylcysteine (NAC) with antibiotic gentamicin was assessed. NAC is a mucolytic agent that has antibacterial properties. It exerts an intense mucolytic-fluidifying action in the mucous and mucopurulent secretions depolymerizing the mucoprotein complexes and nucleic acids responsible of the viscosity to the vitreous and purulent component of the sputum and other secretions. It also has a direct antioxidant action being equipped with a nucleophilic free thiol group (-SH) capable of interacting directly with the electrophilic groups of oxidizing radicals. Zao et al. [19] affirm that the mechanism for the anti-bacterial effect of NAC could be either competitive inhibition of amino-acid (i.e. cysteine) utilization or, reaction with bacterial cell proteins due to a sulfhydryl group in its structure. NAC is widely used through oral, inhalation and intravenous administration and has an excellent safety profile [20]. According to Pérez-Giraldo et al. [21], the action of the NAC on biofilms occurs at concentrations higher than those reached in the blood after intravenous or oral administration, but these concentrations can be achieved through the local application, as shown in our study.

As evidenced by Perry and Neu [22], NAC inhibits the growth of both gram-positive and gram-negative bacteria, including *S. aureus*, *K. pneumoniae* and *P. aeruginosa*. Other authors [16, 19, 21, 23] have pointed out that NAC could decrease biofilm formation of a variety of bacteria and that it inhibites bacterial adherence, reduces the production of extracellular polysaccharide matrix while promoting the disruption of mature biofilms.

In a case of obstructive mumps, Casale et al. [24] used sodium-2-mercaptoethane-sulfonate (Mesna), a mucolytic agent, administered intraductally in association with oral antibiotic-cortisone therapy reporting the resolution of the inflammatory symptoms after the sialolith’s expulsion.

The first to propose intraductal instillation of antibiotics for the treatment of recurrent suppurative parotitis were Quinn and Graham [25] in 1973, which had been successful in all of their 10 patients without recurrence for follow-ups of up to 100 months. They hypothesized that the intraductal antibiotic instillation could reach the microbes remaining in the parenchyma, while systemic antibiotics were adequate for the resolution of acute infections but could act on the purulent material inside the salivary ducts, which remains and causes the subsequent exacerbations.

Intraductal association of broad-spectrum antibiotics and antibiofilm molecules seem to play a certain role in the resolution of infections, the effectiveness of this association on biofilm-producing bacteria has also been highlighted at the cultural level by El-Feky et al. [15] with the use of ciprofloxacin + NAC. Bowling et al. [26] used the intraductal administration of Tetracyline in the Stensen’s duct, exploiting its sclerotic and cytotoxic effects, to obtain the resolution of the symptomatology in chronic recurrent parotitis through atrophy. Antoniades et al. [27] have proposed a comparative study between the use of intraductal instillations of penicillin and saline solution in patients with chronic sialadenitis. They concluded that intraductal instillation of penicillin or saline is a simple and successful technique for the treatment of chronic sialadenitis, and that irrigation itself is the more important factor. Gentamicin, a broad-spectrum aminoglycoside antibiotic that inhibits the protein synthesis of both gram-positive and gram-negative bacteria with bactericide effect, has a certain role in the resolution of the infection. In fact, in association with NAC, the antibiofilm-antibacterial synergistic action probably succeeded where the oral antibiotic alone had failed. Its effectiveness, in association with other molecules, has also been demonstrated in the studies of Sun et al. [28]. As mentioned above, the main limitation of our study was due to the lack of a control group that could allow us to compare the difference in efficacy between the triple intraductal administration and the use of a single drug instillations protocol. However, to address this limitation, we have compared the results obtained from our study and those obtained from a study by Capaccio et al. [10]. The result of this comparison would support the effectiveness of our protocol compared with intraductal steroid-only instillation, in terms of complete symptom remission rate (88.5% vs 55.5%).

Another prospective study by Jokela et al. [29], has shown no significant difference observed in a group of patients receiving 1 mL of hydrocortisone 125 mg/mL and the group receiving 1 mL of isotonic saline solution intraductally, concluding that no additional benefit in symptom relief were found from a single-dose intraductal steroid.

## Conclusion

In our experience, the intraductal administration of gentamicin + NAC + betametasone was used with the aim of resolving the chronic infection which, as we have often seen, is associated with chronic obstructive sialadenitis and often causes relapses even after the endoscopic removal of the obstruction. Our protocol seemed effective also for the therapy of obstructive sialoadenitis where it was not possible to remove or detect endoscopically an obstruction. In all these cases we have noticed an increase in the symptom-free time even in cases where it was not possible to remove the stones.

Given the statistical limitations of our study, further prospective studies of larger case series with longer follow-up as well as case–control studies are needed to establish the possible primary role of gentamicin + NAC + betamethasone intraductal instillation in the treatment for chronic obstructive sialoadenitis.
